# Detecting anteriorly displaced temporomandibular joint discs using super-resolution magnetic resonance imaging: a multi-center study

**DOI:** 10.3389/fphys.2023.1272814

**Published:** 2024-01-05

**Authors:** Yang Li, Wen Li, Li Wang, Xinrui Wang, Shiyu Gao, Yunyang Liao, Yihan Ji, Lisong Lin, Yiming Liu, Jiang Chen

**Affiliations:** ^1^ School and Hospital of Stomatology, Fujian Medical University, Fuzhou, China; ^2^ Fujian Key Laboratory of Oral Diseases, School and Hospital of Stomatology, Fujian Medical University, Fuzhou, China; ^3^ Department of Oral and Maxillofacial Surgery, The First Affiliated Hospital of Zhengzhou University, Zhengzhou, China; ^4^ Department of Oral and Maxillofacial Surgery, The First Affiliated Hospital of Fujian Medical University, Fuzhou, China; ^5^ Department of Oral and Maxillofacial Surgery, Shenzhen Stomatology Hospital, Shenzhen, China; ^6^ School of Mathematics and Statistics, Huazhong University of Science and Technology, Wuhan, China

**Keywords:** transfer learning, temporomandibular joint, MRI, super-resolution, anterior disc displacement

## Abstract

**Background:** Magnetic resonance imaging (MRI) plays a crucial role in diagnosing anterior disc displacement (ADD) of the temporomandibular joint (TMJ). The primary objective of this study is to enhance diagnostic accuracy in two common disease subtypes of ADD of the TMJ on MRI, namely, ADD with reduction (ADDWR) and ADD without reduction (ADDWoR). To achieve this, we propose the development of transfer learning (TL) based on Convolutional Neural Network (CNN) models, which will aid in accurately identifying and distinguishing these subtypes.

**Methods:** A total of 668 TMJ MRI scans were obtained from two medical centers. High-resolution (HR) MRI images were subjected to enhancement through a deep TL, generating super-resolution (SR) images. Naive Bayes (NB) and Logistic Regression (LR) models were applied, and performance was evaluated using receiver operating characteristic (ROC) curves. The model’s outcomes in the test cohort were compared with diagnoses made by two clinicians.

**Results:** The NB model utilizing SR reconstruction with 400 × 400 pixel images demonstrated superior performance in the validation cohort, exhibiting an area under the ROC curve (AUC) of 0.834 (95% CI: 0.763–0.904) and an accuracy rate of 0.768. Both LR and NB models, with 200 × 200 and 400 × 400 pixel images after SR reconstruction, outperformed the clinicians’ diagnoses.

**Conclusion:** The ResNet152 model’s commendable AUC in detecting ADD highlights its potential application for pre-treatment assessment and improved diagnostic accuracy in clinical settings.

## Introduction

As the sole craniomandibular joint in humans and the only left-right joint in the body, temporomandibular joint (TMJ) stands as one of the most complex joints in the human anatomy (Alomar et al., 2007). In contemporary society, temporomandibular disorders (TMD) are becoming increasingly prevalent, affecting the quality of life for approximately 5%–12% of the population (Dworkin et al., 1990; Nishiyama et al., 2012), with a higher incidence among women (Nekora-Azak, 2004). Typically, the disc becomes displaced anteriorly (Oğütcen-Toller et al., 2002), leading to impaired joint function, pain, and other symptoms. The two most prevalent forms of TMJ disc displacement are anterior disc displacement (ADD) with reduction (ADDWR) and ADD without reduction (ADDWoR) (Bas et al., 2012). Clinicians typically conduct a comprehensive clinical examination, which includes the assessment of joint sounds, range of motion, pain patterns, and palpation. Diagnostic imaging, such as magnetic resonance images (MRI), proves invaluable in visualizing the disc’s position and confirming the diagnosis (Limchaichana et al., 2007).

Deep learning (DL) and transfer learning (TL) have demonstrated significant potential in medicine for diagnosing diseases, largely due to their capacity to identify complex patterns within extensive datasets and provide valuable insights for clinical decision-making. DL algorithms can autonomously learn features from raw data, achieving high diagnostic accuracy that frequently surpasses traditional machine learning methods and, in some cases, even human experts (Asch et al., 2019). TL, on the other hand, employs pre-trained models that have already learned features from related tasks, reducing the time and computational resources necessary for training new models (Choudhary et al., 2023). Numerous studies have demonstrated the utilization of DL and TL techniques in the early detection and classification of oral cancers by analyzing histopathological slides and medical images (Bansal et al., 2022). Convolutional Neural Networks (CNNs), a pivotal component of deep learning technology, are designed to extract intricate and discriminative features from input images by employing multi-layered convolution operations (Suzuki, 2017). These extracted features serve as the foundation upon which the model can make precise determinations, ranging from discerning the presence or absence of tumors to performing tumor grading and other complex diagnostic tasks (Truong et al., 2020). Despite notable advancements, current medical imaging radiomics signatures often encounter challenges related to anisotropic resolution and limited voxel statistics (de Farias et al., 2021). To improve the specificity and sensitivity of radiomics models, it is imperative to generate higher resolution images. Super-resolution (SR) technology focuses on enhancing the spatial resolution of digital images derived from lower-resolution observations (Van Reeth et al., 2012). In recent years, the integration of DL techniques has propelled SR to achieve remarkable advancements in the field of medical imaging (Li et al., 2021). Notably, a recent study conducted by Hossein et al. (Mohammad-Rahimi et al., 2023) demonstrated the potential of SR images in enhancing the quality of oral panoramas.

The primary premise of this study is to develop a TL model utilizing SR reconstruction technique MRI data for the diagnosis of ADDWR and ADDWoR. Our aim is to enhance diagnostic efficiency for clinicians through the implementation of this model.

## Methods

### Patients

**FIGURE 1:**
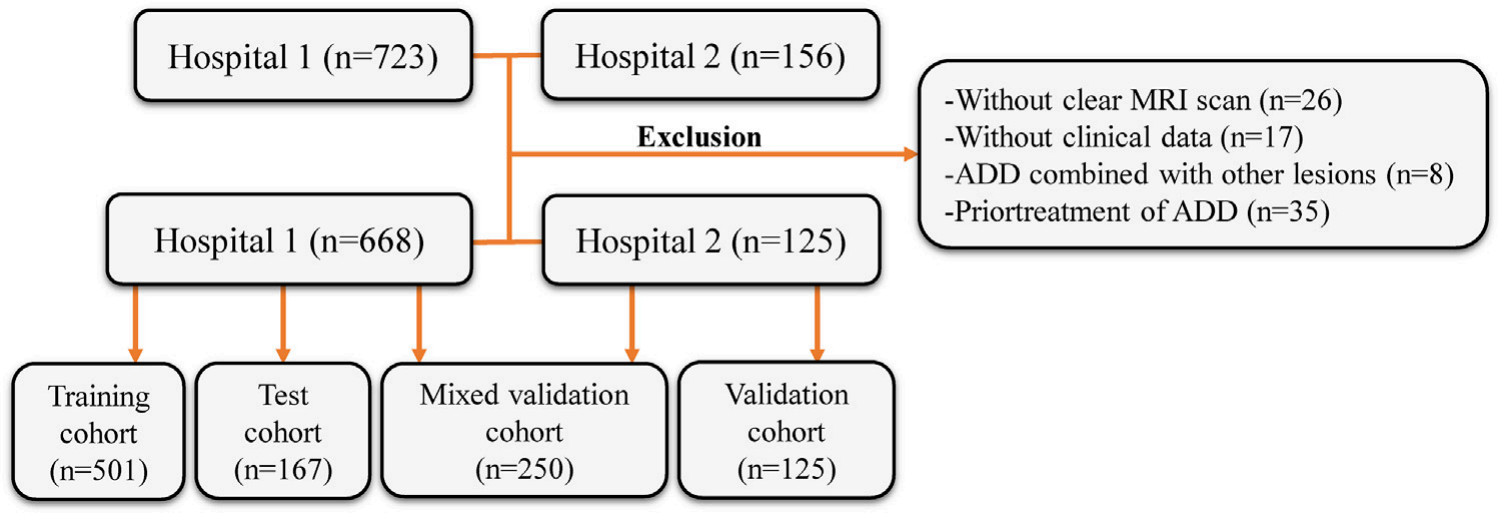
Flow diagram of the study population. MRI, magnetic resonance images; ADD, anterior disc displacement.

This retrospective study’s inclusion criteria and participant recruitment procedures adhered to the guidelines outlined in the 1964 Helsinki Declaration. This retrospective analysis was approved by the ethical review board (2023-KY-0256), and informed consent was waived. The patient registration process is illustrated in Figure 1. This study included patient samples who underwent TMJ MRI examinations at the First Affiliated Hospital of Zhengzhou University from January 2012 to December 2022 and at the First Affiliated Hospital of Fujian Medical University from October 2018 to December 2022. The exclusion criteria included: (Alomar et al., 2007): without clear MRI scan (*n* = 26), (Dworkin et al., 1990), without clinical data (gender, age, etc.) (*n* = 17), (Nishiyama et al., 2012), ADD combined with other lesions (joint disc perforation, joint mass, etc.) (*n* = 8), and (Nekora-Azak, 2004) prior treatment of ADD (conservative treatment or surgical treatment) (*n* = 35). In accordance with the sequence of patient examinations, a total of 668 MRI images from the First Affiliated Hospital of Zhengzhou University were partitioned into two distinct groups: the training cohort (*n* = 501) and the test cohort (*n* = 167). MRI images from the First Affiliated Hospital of Fujian Medical University were the validation cohort (*n* = 125). Simultaneously, 125 MRI images from the test cohort and 125 MRI images from the validation cohort were randomly chosen to create a new mixed validation cohort. This cohort was employed to assess the performance of the model by incorporating mixed multicenter data for evaluation.

### Image acquisition and processing

The First Affiliated Hospital of Zhengzhou University consistently employs a 3T superconducting magnetic resonance scanner (Siemens, Germany) along with a TMJ surface coil. In this study, T2-weighted imaging (T2WI) sequence images were selected for analysis. The parameters are as follows: oblique sagittal plane T2WI: TR 2,000 ms, TE 76 ms, FOV 256 mm × 256 mm. The thickness for the above sequence layers is 3 mm. Similarly, the First Affiliated Hospital of Fujian Medical University utilizes a 3T superconducting magnetic resonance scanner (Siemens, Germany) and a TMJ surface coil. Scanning parameters include: oblique sagittal plane T2WI: TR 4,000 ms, TE 79 ms, FOV 220 mm × 220 mm. The thickness for the above sequence layers is 5 mm.

Employing the widely-used MRI oblique sagittal reading method, oblique sagittal images of open position was selected for evaluation (Orhan et al., 2021). Based on the position of the posterior band of the joint disc and the condyle in the open position, patients were judged to have either ADDWR (posterior band located anterior to the condylar head in the closed-mouth position but with a normal disc-condyle relationship in the open-mouth position) or ADDWoR (posterior band positioned anterior to the condyle in both the closed-mouth and open-mouth positions). All MRI image assessments in this study were conducted by two highly experienced physicians in the imaging department, each specializing in diagnosing TMD for over 10 years. In the event of any disagreement in the diagnosis, a senior imaging physician with over 30 years of experience in diagnosing TMD will make the final judgment.

We standardized the preprocessing methodology for all images acquired from two distinct medical centers. Initially, we employed a resampling technique on the selected MRI images, adjusting the pixel dimensions to 1 mm × 1 mm × 1 mm (Huang et al., 2023). This resampling procedure ensured the preservation of spatial consistency across images possessing varying resolutions. Subsequently, we applied a standardization process to the resampled images, harmonizing the parameters of the two medical centers of images to conform to a unified standard (Nyúl et al., 2000). Images were manually selected, ensuring the condyle was visible and positioned within 1/3 of the image center. To avoid any potential impact on model recognition, the image size was reduced to improve training efficiency. The images were cropped to 50 × 50 pixel and 100 × 100 pixel. Building upon the acquired high-resolution (HR) images, we employ a deep DL network to enhance the longitudinal resolution by quadrupling the pixel count. First, Gaussian noise was added to the MRI to reduce the out-plane resolution with a factor of four to generate a new low-resolution image. Then, the low-resolution and synthetic HR image pairs were used to train a lightweight parallel generative adversarial network (GAN) model. Finally, the trained model was applied to HR by TL. Consequently, the resulting images are referred to as SR images.

### Feature extraction

**FIGURE 2:**
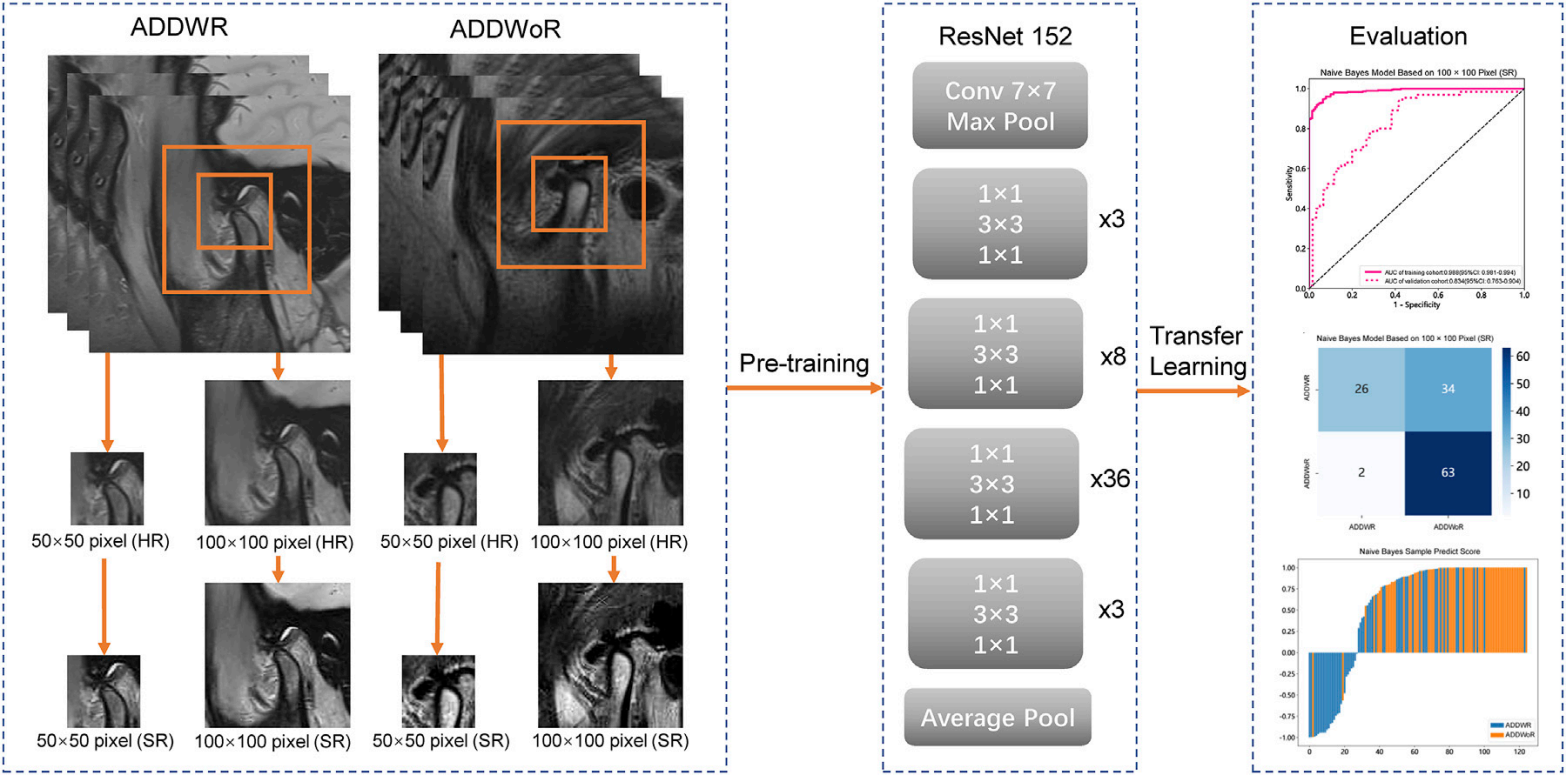
Workflow of the study. ADDWR, anterior disc displacement with reduction; ADDWoR, anterior disc displacement without reduction; HR, high-resolution; SR, super-resolution.

The model process is depicted in Figure 2. The workflow employed in this study involved the input of cropped images into three CNN models (ResNet152, DenseNet201 and GoogLeNet) separately, which, in turn, facilitated the extraction of salient image features through TL. Following this feature extraction step, our investigation leveraged two distinct machine learning models: Naive Bayes (NB) and Logistic Regression (LR). The primary objective was to meticulously assess and quantify the performance of the model under scrutiny. The CNN model, which was pre-trained on the ImageNet dataset, was used for TL. On one hand, the HR image with pixel dimensions of 50 × 50 and 100 × 100 is inputted into the model structure. On the other hand, the above two groups of images with different pixels are subjected to SR reconstruction. The obtained images with 200 × 200 and 400 × 400 pixel are then input into the same structure. These HR and SR images serve as the original inputs for four distinct groups of models. Model training was performed by updating the network weights using a cross-entropy loss function for the prediction task. An adaptive moment estimation optimizer was implemented with a learning rate of 0.1 for 30 epochs using a batch size of 64. The network parameters were fixed after training was completed and the fixed model was used as a feature extractor. Finally, DL features were extracted from the penultimate layer of the fine-tuned model for each patient in the training and validation cohorts. The model was subsequently assessed visually to comprehend its functioning. The classification activation heatmap was employed to emphasize the most crucial anatomical regions utilized by the model when categorizing images as ADDWR and ADDWoR. Classification activation heatmaps are heat gradient maps that employ warmer colors to indicate regions of greater significance in the classification process.

### Feature selection

In this study, the Mann-Whitney U test and feature screening were conducted on all DL features. Only features with *p* < 0.05 were considered for further analysis. Spearman’s rank correlation coefficient was employed to assess the correlation between features with high repeatability. To avoid redundancy, only one feature from any pair exhibiting a correlation coefficient greater than 0.9 was retained (Wang et al., 2021). In order to maximize the informative value of the feature set, a greedy recursive deletion strategy was employed for feature filtering (Farahat et al., 2013). The Least Absolute Shrinkage and Selection Operator (LASSO) regression model was utilized to construct a signature based on the discovery dataset. LASSO regression shrinks all regression coefficients towards zero and sets many coefficients of uncorrelated features to exactly zero. The optimal regularization weight λ was determined using a minimum criterion and 10-fold cross-validation. After LASSO feature screening, the final features were input into machine learning models, including LR and NB for constructing the model.

### Comparison of DL model and clinicians’ diagnostic performance

Two oral and maxillofacial clinicians were selected, one with 3 years of experience and the other with 5 years. Subsequently, the MRI images of the test cohort were entrusted to these two clinicians for evaluation. This training encompassed the identification and diagnosis of ADDWR and ADDWoR from oblique sagittal images obtained from SR-processed MRI scans. Subsequently, the two clinicians participated in the training session and applied their acquired knowledge to diagnose the disease within the test cohort. Notably, the test cohort consisted of 167 oblique sagittal MRI images that had undergone SR processing. It is important to note that these 167 images in the test cohort were distinct from those in the training cohort.

### Statistical analysis

The predictive power of NB and LR models was assessed using receiver operating characteristic (ROC) curves; the area under the ROC curve (AUC) was calculated and the balanced sensitivity and specificity of the cut-off point giving the maximum value of the Youden index was calculated. The 95% confidence interval (CI) of AUC was calculated using the bootstrap method (1,000 intervals). The AUC ranges from 0.5 to 1.0 and serves as a metric for evaluating the discriminative power of a test. An AUC value of 1.0 indicates a perfect discriminant test, while an AUC ranging from 0.8 to 1.0 signifies a good discriminant test. In cases where the AUC falls between 0.6 and 0.8, the discriminant test is considered moderate. However, if the AUC ranges from 0.5 to 0.6, the discriminant test is regarded as poor (Weinstein et al., 1980; Meehan et al., 2022). Statistical analyses were performed using SPSS software (version 21.0). Statistical significance was defined as a two-sided *p* ≤ 0.05.

Sensitivity, specificity, and accuracy were computed to evaluate the performance of the classification model. The definitions are as follows: where TP (true positives) and TN (true negatives) represent correct classifications, while FP (false positives) and FN (false negatives) indicate incorrect classifications.

$$Sensitivity=\frac{TP}{TP+FN}$$

$$Specificity=\frac{TN}{TN+FP}$$

$$Accuracy=\frac{TP+TN}{TP+FN+FP+TN}$$

## Results

### Patient characteristics

A total of 391 patients, encompassing 668 TMJs, were identified in the First Affiliated Hospital of Zhengzhou University. Among these, 295 patients with a mean age of 30.31 ± 16.14 (age range of 11–90 years, 45 males, 250 females) and 501 MRI images were selected as the training cohort, while 96 patients with a mean age of 30.75 ± 16.04 (age range of 12–70 years, 10 males, 86 females) and 167 MRI images were chosen as the test cohort. The First Affiliated Hospital of Fujian Medical University contributed a total of 96 patients with a mean age of 25.90 ± 10.63 (age range of 12–68 years, 19 males, 77 females) and 125 MRI images for the validation cohort.

### Model comparation

**FIGURE 3:**
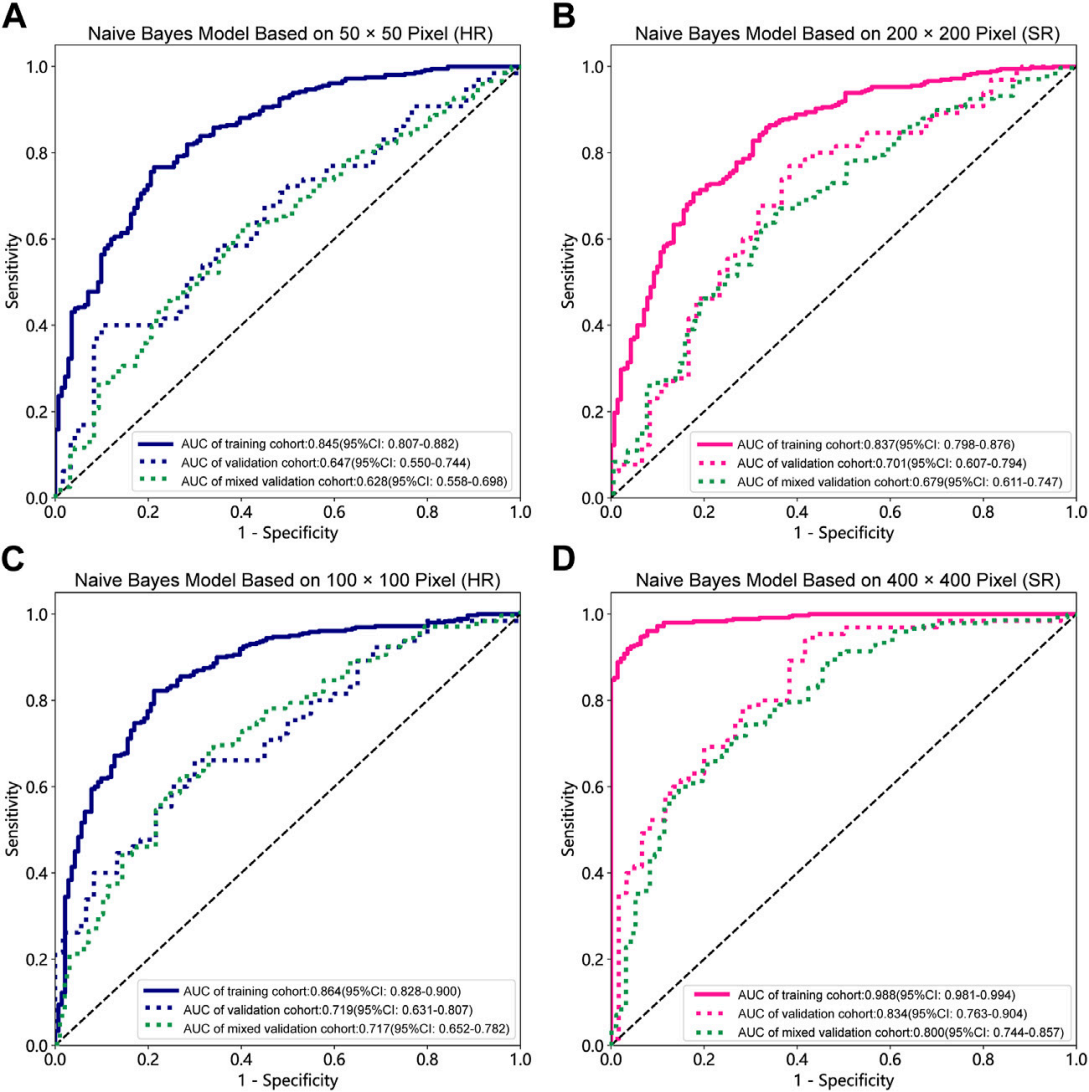
Receiver operating characteristic (ROC) curves of the different pixel in the Naive Bayes models (ResNet152). **(A)** 50 × 50 pixel images (HR). **(B)** 200 × 200 pixel images (SR). **(C)** 100 × 100 pixel images (HR). **(D)** 400 × 400 pixel images (SR). AUC, area under ROC curve; HR, high-resolution; SR, super-resolution.

**FIGURE 4:**
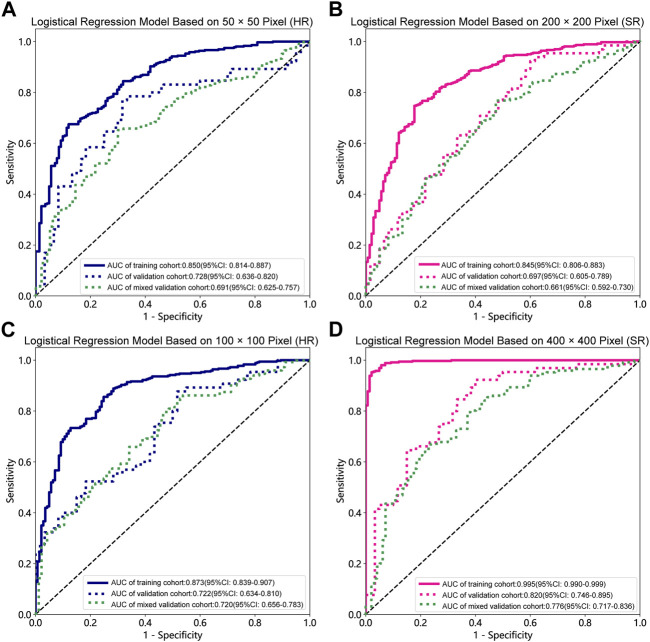
Receiver operating characteristic (ROC) curves of the different pixel in the Logistic Regression models (ResNet152). **(A)** 50 × 50 pixel images (HR). **(B)** 200 × 200 pixel images (SR). **(C)** 100 × 100 pixel images (HR). **(D)** 400 × 400 pixel images (SR). AUC, area under ROC curve; HR, high-resolution; SR, super-resolution.

**FIGURE 5:**
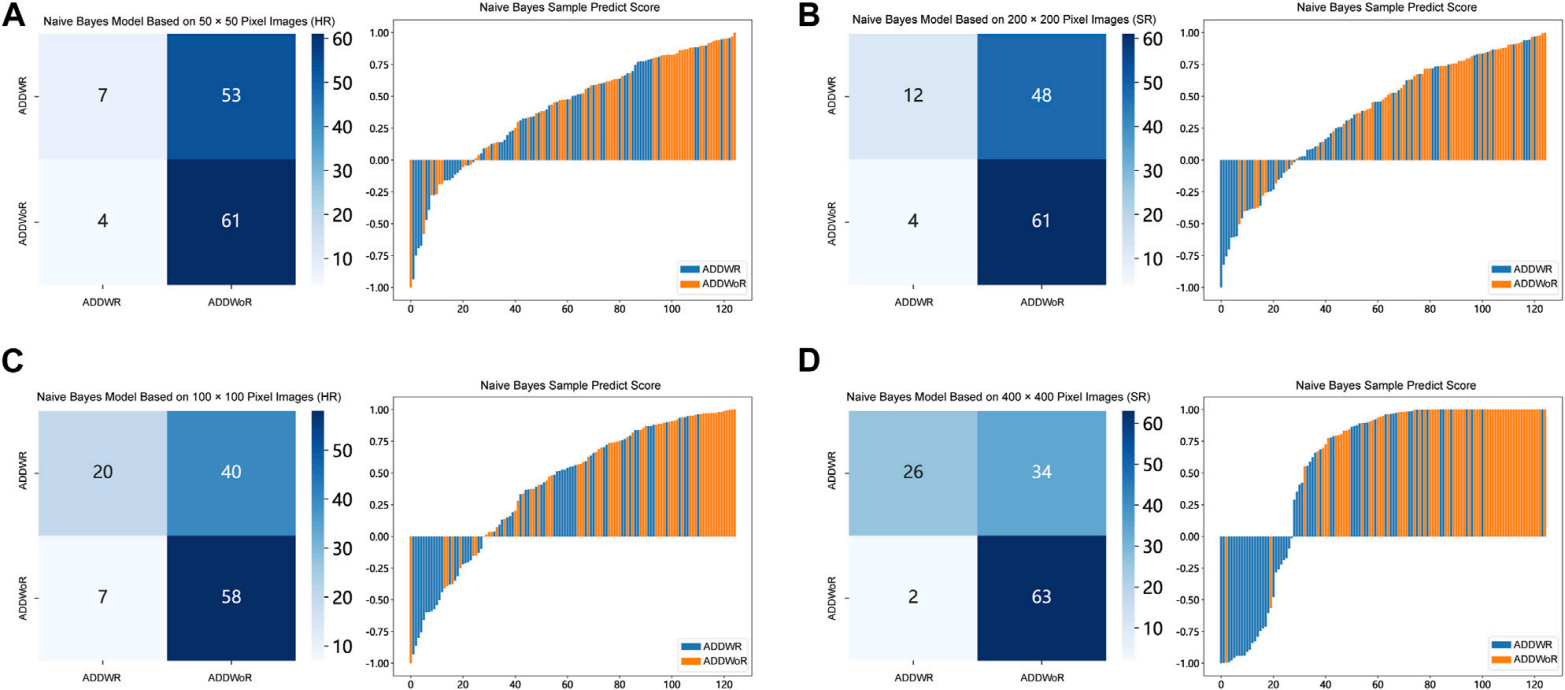
Confusion matrix and sample prediction histogram in the Naive Bayes models of different pixel images (ResNet152) (Validation cohort). **(A)** 50 × 50 pixel images (HR). **(B)** 200 × 200 pixel images (SR). **(C)** 100 × 100 pixel images (HR). **(D)** 400 × 400 pixel images (SR). ADDWR: anterior disc displacement with reduction; ADDWoR, anterior disc displacement without reduction; HR, high-resolution; SR, super-resolution.

**FIGURE 6:**
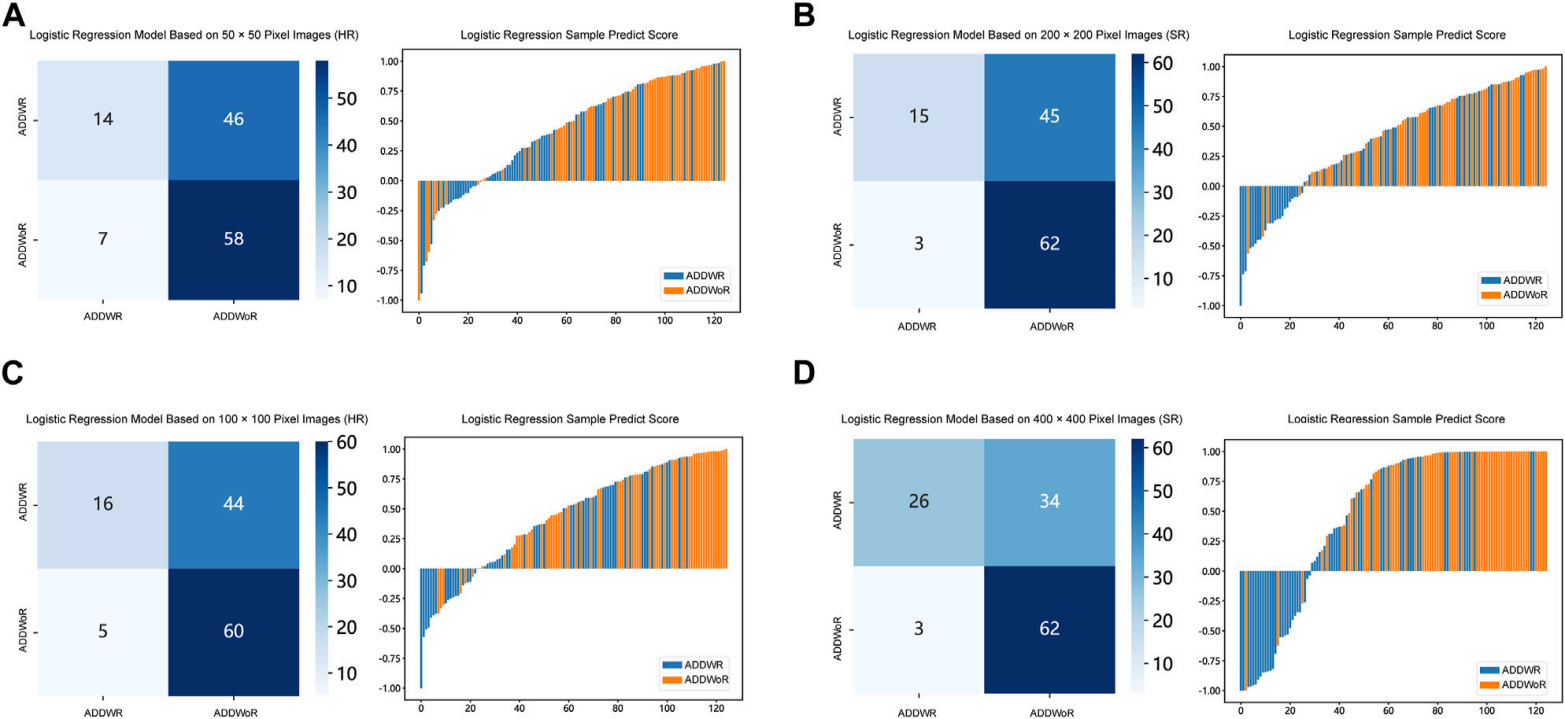
Confusion matrix and sample prediction histogram in the Logistic Regression models of different pixel images (ResNet152) (Validation cohort). **(A)** 50 × 50 pixel images (HR). **(B)** 200 × 200 pixel images (SR). **(C)** 100 × 100 pixel images (HR). **(D)** 400 × 400 pixel images (SR). ADDWR: anterior disc displacement with reduction; ADDWoR, anterior disc displacement without reduction; HR, high-resolution; SR, super-resolution.

Table 1, Table 2 and Supplementary Tables S1–S4 summarize the performance of NB and LR models utilizing TL based on ResNet152, DenseNet201 and GoogLeNet separately. The training and validation cohort’s ROC and AUC with 95% CI of NB and LR models are provided in Figure 3, Figure 4 and Supplementary Figures S1–S4. The mixed validation cohort’s ROC and AUC with 95% CI of NB and LR models based on ResNet152 are provided in Table 1 and Table 2. Between the models that compared the input of four different types of images, the validation cohort demonstrated superior performance for the 400 × 400 pixel model utilizing SR reconstruction based on ResNet152 with the NB algorithm. The AUC of this model reached 0.834 (95% CI: 0.763–0.904), accompanied by an accuracy rate of 0.768. The next step of performance evaluation was conducted for the ResNet152 model. Figure 5 showcases the confusion matrix and histogram of predicted outcomes for each patient within the NB model groups using the four types of images, alongside their corresponding true outcomes. Furthermore, Figure 6 presents the comparative results relative to the LR model. Supplementary Figures S5, S6 presents the comparative results based on mixed validation cohort.

**TABLE 1:** Performance measures in Naive Bayes models of two different pixels for images based on validation cohort (ResNet152).

Type	Task	Accuracy	Sensitivity	Specificity	AUC	95% CI
HR 50 × 50	Training cohort	0.772	0.767	0.787	0.845	0.807–0.882
	Validation cohort	0.640	0.400	0.900	0.647	0.550–0.744
	Mixed validation cohort	0.620	0.598	0.634	0.628	0.558–0.698
SR 200 × 200	Training cohort	0.739	0.706	0.823	0.837	0.798–0.876
	Validation cohort	0.696	0.769	0.617	0.701	0.607–0.794
	Mixed validation cohort	0.656	0.639	0.671	0.679	0.611–0.747
HR 100 × 100	Training cohort	0.812	0.822	0.787	0.864	0.828–0.900
	Validation cohort	0.680	0.662	0.700	0.719	0.631–0.807
	Mixed validation cohort	0.680	0.670	0.686	0.717	0.652–0.782
SR 400 × 400	Training cohort	0.932	0.919	0.965	0.988	0.981–0.994
	Validation cohort	0.768	0.938	0.583	0.834	0.763–0.904
	Mixed validation cohort	0.700	0.856	0.601	0.800	0.744–0.857

HR, high-resolution; SR, super-resolution; AUC, area under ROC, curve; CI, confidence interval; ROC, receiver operating characteristic.

**TABLE 2:** Performance measures in logistic regression models of two different pixels for images based on validation cohort (ResNet152).

Type	Task	Accuracy	Sensitivity	Specificity	AUC	95% CI
HR 50 × 50	Training cohort	0.733	0.675	0.879	0.850	0.814–0.887
	Validation cohort	0.728	0.769	0.683	0.728	0.636–0.820
	Mixed validation cohort	0.672	0.701	0.654	0.691	0.625–0.757
SR 200 × 200	Training cohort	0.768	0.747	0.823	0.845	0.806–0.883
	Validation cohort	0.672	0.923	0.400	0.697	0.605–0.789
	Mixed validation cohort	0.672	0.526	0.770	0.661	0.592–0.730
HR 100 × 100	Training cohort	0.826	0.856	0.752	0.873	0.839–0.907
	Validation cohort	0.688	0.877	0.483	0.722	0.634–0.810
	Mixed validation cohort	0.696	0.536	0.797	0.720	0.656–0.783
SR 400 × 400	Training cohort	0.960	0.953	0.979	0.995	0.990–0.999
	Validation cohort	0.768	0.923	0.600	0.820	0.746–0.895
	Mixed validation cohort	0.704	0.784	0.654	0.776	0.717–0.836

HR, high-resolution; SR, super-resolution; AUC, area under ROC, curve; CI, confidence interval; ROC, receiver operating characteristic.

### Classification activation heatmap

**FIGURE 7:**
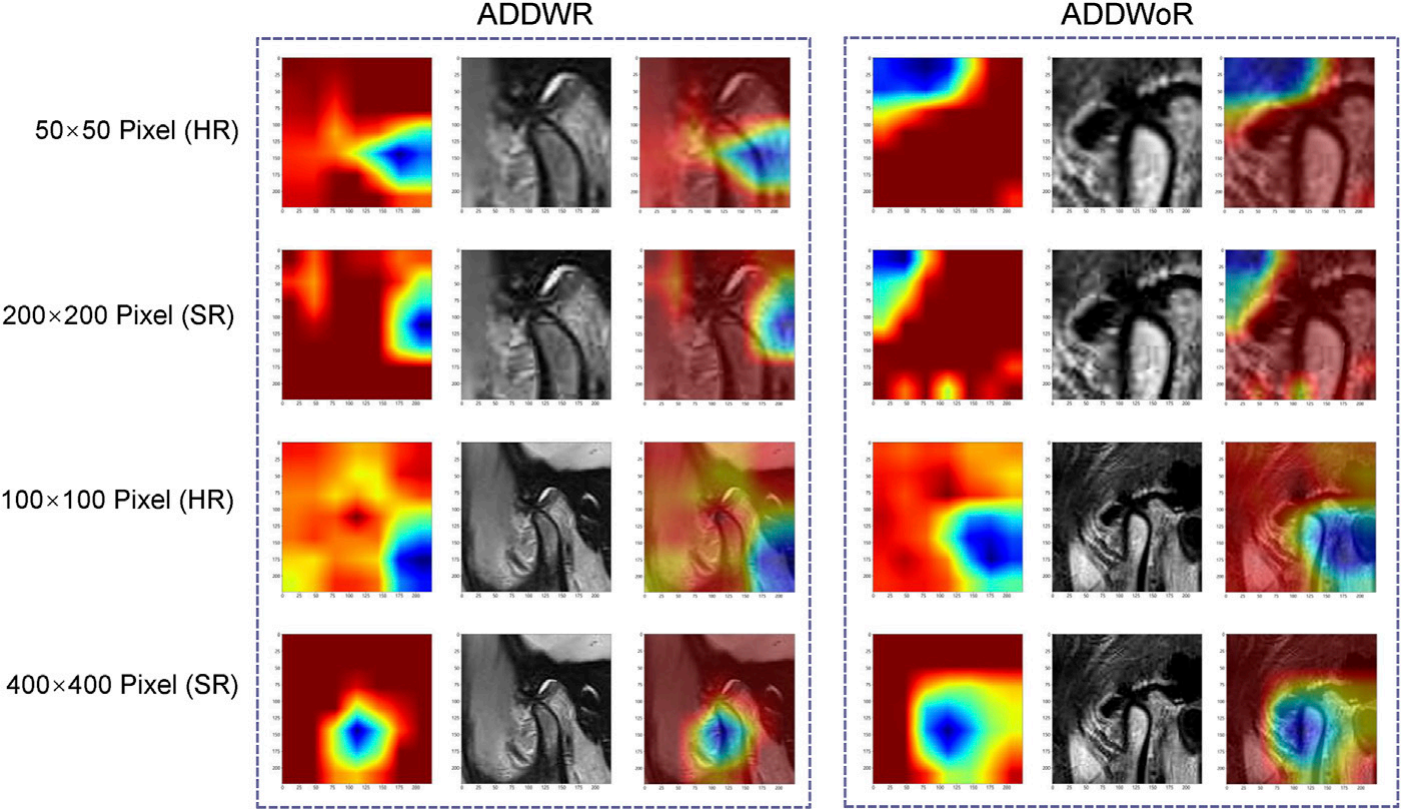
Classification activation heatmap. ADDWR, anterior disc displacement with reduction; ADDWoR, anterior disc displacement without reduction; HR, high-resolution; SR, super-resolution.

Figure 7 depicts the class activation maps for the four types of image pixel. The red color on the heatmap represents the most influential area, while the blue color signifies the least influential area in the decision-making process. It is evident that in the 400 × 400 pixel image post SR reconstruction, the mandibular condyle serves as the reference point, with the surrounding regions being identified as significant areas.

### The results of test cohort

Table 3 demonstrates that the AUC of both NB and LR models, with pixel of 200 × 200 and 400 × 400 pixel images after SR reconstruction, surpass those of the two clinicians. Specifically, the AUC for the NB model with 400 × 400 pixel image after SR reconstruction technology is 0.754 (95% CI: 0.666–0.842), indicating moderate performance. However, the AUC for the LR with 400 × 400 pixel image after SR reconstruction technology slightly decreases to 0.743 (95% CI: 0.653–0.834). Furthermore, the AUC for the 200 × 200 pixel image reconstructed using SR is even lower. Notably, the AUC values for the 50 × 50 and 100 × 100 pixel images interpreted by the two clinicians show minimal disparity.

**TABLE 3:** Performance measures in Naive Bayes and logistic regression models for test cohort SR images, compared with judgments by oral and maxillofacial clinicians (ResNet152).

Type	Accuracy (%)	Sensitivity (%)	Specificity (%)	AUC	95% CI
LR 200 × 200	0.671	0.636	0.761	0.711	0.625–0.798
LR 400 × 400	0.695	0.678	0.739	0.743	0.653–0.834
NB 200 × 200	0.653	0.595	0.804	0.707	0.622–0.793
NB 400 × 400	0.713	0.694	0.761	0.754	0.666–0.842
Clinician I 200 × 200	0.509	0.543	0.496	0.520	0.422–0.618
Clinician I 400 × 400	0.551	0.500	0.570	0.535	0.437–0.633
Clinician II 200 × 200	0.713	0.810	0.457	0.633	0.534–0.732
Clinician II 400 × 400	0.755	0.868	0.457	0.662	0.563–0.761

LR, logistic regression; NB, naive bayes; AUC, area under ROC, curve; CI, confidence interval; ROC, receiver operating characteristic.

## Discussion

TMD encompass a group of conditions involving pain and dysfunction of the TMJ, masticatory muscles, and surrounding structures. An increasing number of studies have discovered that the disorder progressively results in emotional and psychological disturbances (Sójka et al., 2019), negatively impacting patients’ quality of life (Tjakkes et al., 2010). ADD represents a subgroup of TMD and is one of the most common TMD. It occurs when the articular disc, a fibrocartilaginous structure situated between the mandibular condyle and the temporal bone, becomes displaced forward relative to the condyle. ADD may lead to osteoarthritis, joint ankylosis, and reduced condylar height (Zhuo et al., 2015), potentially affecting mandibular development or causing mandibular asymmetry.

MRI serves as a non-invasive imaging modality, offering valuable insights into the soft tissues of the TMJ, such as the articular disc, ligaments, and surrounding muscle tissue. In ADDWoR, the joint space may appear narrower and the condyle may be situated more anteriorly, while in ADDWR, the joint space and condyle position exhibit a more normal appearance during mouth opening. Consequently, by selecting MRI images of the patient’s oral opening position for disease diagnosis, clinicians can differentiate between these two types of ADD, thereby supporting the development of subsequent individualized and tailored treatment plans, as well as predicting the prognosis of TMD (Ahmad et al., 2009). The application of the diagnostic model developed in this study has the potential to significantly enhance the diagnostic accuracy and efficiency of physicians in clinical practice. By leveraging the model’s capabilities, clinicians may experience improved accuracy in identifying TMJ anterior displacement conditions, namely, ADDWR and ADDWoR. Additionally, the integration of this model into clinical workflows has the potential to mitigate the time-consuming process of manually interpreting MRI films.

In this study, a total of 793 TMJs from 487 patients, spanning two hospitals in southern and northern China. The diverse and representative patient sample increases the potential for generalizability of the study’s results to a broader population. Simultaneously, the multi-center data not only reduces the risk of overfitting but also bolsters confidence in the model’s ability to generalize to new, unseen data (Falconieri et al., 2020). Referring to Tables 1, 2, the comparison demonstrates that there is no significant disparity in the model’s performance evaluation between validation cohort and mixed validation cohort. Our inference is that following the resampling and standardization procedures, the distinctions observed in the multi-center imaging data appear to exert minimal influence on the model’s performance.

DL models have been employed for the identification of TMD. Jung et al. (2023) utilized a TL model to classify images of normal TMJs and osteoarthritis, and their results demonstrated the model’s effectiveness in recognizing osteoarthritis. Choi et al.’s research (Choi et al., 2021) indicates that image-based artificial intelligence models for diagnosing TMJ osteoarthritis offer diagnostic performance comparable to that of oral and maxillofacial radiology experts, suggesting that this model can play a crucial role in most clinics lacking such experts. However, there are currently limited reports on the application of SR reconstruction technology in the diagnosis of TMD. In the SR-processed 200 × 200 pixel group and 400 × 400 pixel group, the image resolution has been enhanced fourfold, resulting in finer texture and sharper edges. Upon analyzing the feature extraction outcomes of this image set, the NB model demonstrated superior performance. Comparing these results with those obtained without super-resolution reconstruction, it is observed that the AUC of the 200 × 200 pixel group, despite using SR technology, did not exhibit significant improvement. However, in the 400 × 400 pixel group, the AUC value increased from 0.719 to 0.834, signifying a notable enhancement from a moderate to a good performance level. Furthermore, the model’s confusion matrix and histogram of prediction results exemplify the ideal diagnostic effect. NB offers computational efficiency and the ability to effectively handle high-dimensional features extracted by DL models, resulting in faster training and inference times while simplifying complex representation processing (Singh et al., 2017). Comparing the models constructed using the four types of pixel collectively, it is evident that the AUC value of the 100 × 100 pixel group consistently surpasses that of the 50 × 50 pixel group. This finding implies that larger image pixel offers enhanced resolution and finer details, thereby aiding DL algorithms in capturing underlying patterns and features within the data more effectively (Cha et al., 2017). Moreover, the utilization of larger image pixel enables CNN to extract a more extensive range of diverse and discriminative features, empowering models to learn from richer datasets (Mopuri et al., 2018).

Within the 400 × 400 pixel group, which has undergone SR reconstruction, the categorical activation heatmaps of the two samples (ADDWR and ADDWoR, respectively) exhibit more precise focus regions. This improved accuracy can be attributed to the utilization of SR-processed 400 × 400 pixel groups, which offer the model more comprehensive and detailed information. Consequently, the model can better prioritize the most relevant and discriminative features within the image (Dusmanu et al., 2019).

In predicting ADD classification, the ROC of the model ranks higher than two clinicians when comparing a ResNet152 model based on DL to trained clinicians. In terms of specificity, the ResNet152 model performs exceptionally well and surpasses that of the clinicians. High specificity models can make the diagnosis of TMD more effective. Since clinicians demonstrate higher sensitivity in determining the presence or absence of ADD, the appropriate use of these machines by clinicians could improve diagnostic accuracy.

The novelty of this article resides in the utilization of advanced techniques such as deep transfer learning and super-resolution reconstruction within the context of ADD diagnosis, thereby enhancing the efficiency of model evaluation. Significantly, this study presents advancements in various aspects compared to prior research, including enhancements in sample selection, data preprocessing, and model construction. However, our research, while promising, is not without its limitations. Firstly, the current system’s capabilities are confined solely to recognizing ADD, with no capacity to identify the relatively infrequent instances of posterior and lateral displacement frequently encountered in clinical practice. Addressing this limitation necessitates an expansion of our dataset to encompass a more comprehensive array of TMJ images. We are committed to this pursuit and intend to develop a multi-classification model, empowered by DL, that can aptly discern and categorize various forms of TMJ displacement. Secondly, we acknowledge that our study, though enriched by data from two medical centers, was restricted by the limited number of images employed for model training. Amplifying the size of our dataset, through the incorporation of more diverse images, is a logical next step that promises to enhance the model’s overall performance. Additionally, we recognize that our current model’s scope is confined to the analysis of a single sagittal MRI image. Nevertheless, it is crucial to underscore that a substantial reservoir of valuable information is embedded within the coronal images. Hence, our future research endeavors will entail the development of advanced DL models capable of comprehensively recognizing and interpreting all image orientations.

## Conclusion

In this investigation, we conducted a rigorous analysis of SR reconstruction applied to MRI images obtained from patients diagnosed with ADD of the TMJ. Through a meticulous comparative analysis of various models, our findings elucidate a notable trend: the SR reconstruction using 400 × 400 pixel group, in conjunction with the NB model, yielded superior performance outcomes. SR reconstruction, as exemplified by our investigation, represents an innovative approach that transcends the realm of MRI images. Indeed, this technique bears the potential to revolutionize the efficiency of diverse medical imaging modalities and, more significantly, facilitate the seamless translation of cutting-edge technology into the intricate landscape of clinical practice.

## Data Availability

The original contributions presented in the study are included in the article/Supplementary Material, further inquiries can be directed to the corresponding authors.
