# Supplementary material for: Detecting anteriorly displaced temporomandibular joint discs using super-resolution magnetic resonance imaging: a multi-center study
Source: Front Physiol. 2024 Jan 5;14:1272814. doi: 10.3389/fphys.2023.1272814 (PMC10796555; doi:10.3389/fphys.2023.1272814)
Supplement: Supplementary file 1 [file DataSheet1.docx]

Supplementary Material

**Detecting anteriorly displaced temporomandibular joint discs using super-resolution magnetic resonance imaging: a multi-center study**

# Supplementary Tables

**Supplementary Table 1.** Performance measures in Naive Bayes models of two different pixels for images based on validation cohort (DenseNet201).

| **Type** | **Task** | **Accuracy** | **Sensitivity** | **Specificity** | **AUC** | **95% CI** |
| --- | --- | --- | --- | --- | --- | --- |
| **HR 50**×**50** | Training cohort | 0.858 | 0.822 | 0.950 | 0.950 | 0.931 - 0.969 |
|  | Validation cohort | 0.672 | 0.646 | 0.712 | 0.710 | 0.620 - 0.800 |
| **SR 200**×**200** | Training cohort | 0.852 | 0.883 | 0.773 | 0.898 | 0.868 - 0.928 |
|  | Validation cohort | 0.664 | 0.862 | 0.450 | 0.662 | 0.565 - 0.759 |
| **HR 100**×**100** | Training cohort | 0.944 | 0.942 | 0.950 | 0.985 | 0.975 - 0.995 |
|  | Validation cohort | 0.656 | 0.692 | 0.617 | 0.712 | 0.623 - 0.801 |
| **SR 400**×**400** | Training cohort | 0.766 | 0.728 | 0.865 | 0.867 | 0.833 - 0.901 |
|  | Validation cohort | 0.632 | 0.462 | 0.817 | 0.689 | 0.597 - 0.781 |

HR: high-resolution; SR: super-resolution; AUC: area under ROC curve; CI: confidence interval; ROC: receiver operating characteristic.

**Supplementary Table 2.** Performance measures in Logistic Regression models of two different pixels for images based on validation cohort (DenseNet201).

| **Type** | **Task** | **Accuracy** | **Sensitivity** | **Specificity** | **AUC** | **95% CI** |
| --- | --- | --- | --- | --- | --- | --- |
| **HR 50**×**50** | Training cohort | 0.908 | 0.911 | 0.901 | 0.963 | 0.948 - 0.979 |
|  | Validation cohort | 0.680 | 0.677 | 0.683 | 0.726 | 0.639 - 0.814 |
| **SR 200**×**200** | Training cohort | 0.862 | 0.875 | 0.830 | 0.919 | 0.891 - 0.946 |
|  | Validation cohort | 0.704 | 0.708 | 0.700 | 0.721 | 0.632 - 0.810 |
| **HR 100**×**100** | Training cohort | 0.972 | 0.981 | 0.950 | 0.993 | 0.988 - 0.998 |
|  | Validation cohort | 0.672 | 0.938 | 0.383 | 0.658 | 0.561 - 0.754 |
| **SR 400**×**400** | Training cohort | 0.818 | 0.819 | 0.816 | 0.881 | 0.850 - 0.912 |
|  | Validation cohort | 0.712 | 0.600 | 0.833 | 0.743 | 0.655 - 0.830 |

HR: high-resolution; SR: super-resolution; AUC: area under ROC curve; CI: confidence interval; ROC: receiver operating characteristic.

**Supplementary Table 3.** Performance measures in Naive Bayes models of two different pixels for images based on validation cohort (GoogLeNet).

| **Type** | **Task** | **Accuracy** | **Sensitivity** | **Specificity** | **AUC** | **95% CI** |
| --- | --- | --- | --- | --- | --- | --- |
| **HR 50**×**50** | Training cohort | 0.713 | 0.714 | 0.709 | 0.769 | 0.722 - 0.816 |
|  | Validation cohort | 0.600 | 0.400 | 0.817 | 0.604 | 0.504 - 0.703 |
| **SR 200**×**200** | Training cohort | 0.699 | 0.686 | 0.730 | 0.745 | 0.697 - 0.793 |
|  | Validation cohort | 0.600 | 0.908 | 0.271 | 0.572 | 0.469 - 0.674 |
| **HR 100**×**100** | Training cohort | 0.729 | 0.733 | 0.716 | 0.784 | 0.740 - 0.828 |
|  | Validation cohort | 0.608 | 0.800 | 0.407 | 0.542 | 0.438 - 0.646 |
| **SR 400**×**400** | Training cohort | 0.737 | 0.781 | 0.624 | 0.749 | 0.700 - 0.798 |
|  | Validation cohort | 0.560 | 0.908 | 0.186 | 0.513 | 0.410 - 0.616 |

HR: high-resolution; SR: super-resolution; AUC: area under ROC curve; CI: confidence interval; ROC: receiver operating characteristic.

**Supplementary Table 4.** Performance measures in Logistic Regression models of two different pixels for images based on validation cohort (GoogLeNet).

| **Type** | **Task** | **Accuracy** | **Sensitivity** | **Specificity** | **AUC** | **95% CI** |
| --- | --- | --- | --- | --- | --- | --- |
| **HR 50**×**50** | Training cohort | 0.685 | 0.678 | 0.702 | 0.719 | 0.668 - 0.770 |
|  | Validation cohort | 0.664 | 0.523 | 0.831 | 0.677 | 0.583 - 0.772 |
| **SR 200**×**200** | Training cohort | 0.679 | 0.692 | 0.645 | 0.707 | 0.655 - 0.758 |
|  | Validation cohort | 0.592 | 0.892 | 0.267 | 0.527 | 0.424 - 0.631 |
| **HR 100**×**100** | Training cohort | 0.760 | 0.828 | 0.589 | 0.760 | 0.712 - 0.808 |
|  | Validation cohort | 0.560 | 0.800 | 0.305 | 0.530 | 0.428 - 0.632 |
| **SR 400**×**400** | Training cohort | 0.731 | 0.781 | 0.624 | 0.749 | 0.703 - 0.796 |
|  | Validation cohort | 0.560 | 0.908 | 0.186 | 0.500 | 0.397 - 0.603 |

HR: high-resolution; SR: super-resolution; AUC: area under ROC curve; CI: confidence interval; ROC: receiver operating characteristic.

**Supplementary Figure 1.** Receiver operating characteristic (ROC) curves of the different pixel in the Naive Bayes models (DenseNet201). A: 50 × 50 pixel images (HR). B: 200 × 200 pixel images (SR). C: 100 × 100 pixel images (HR). D: 400 × 400 pixel images (SR).


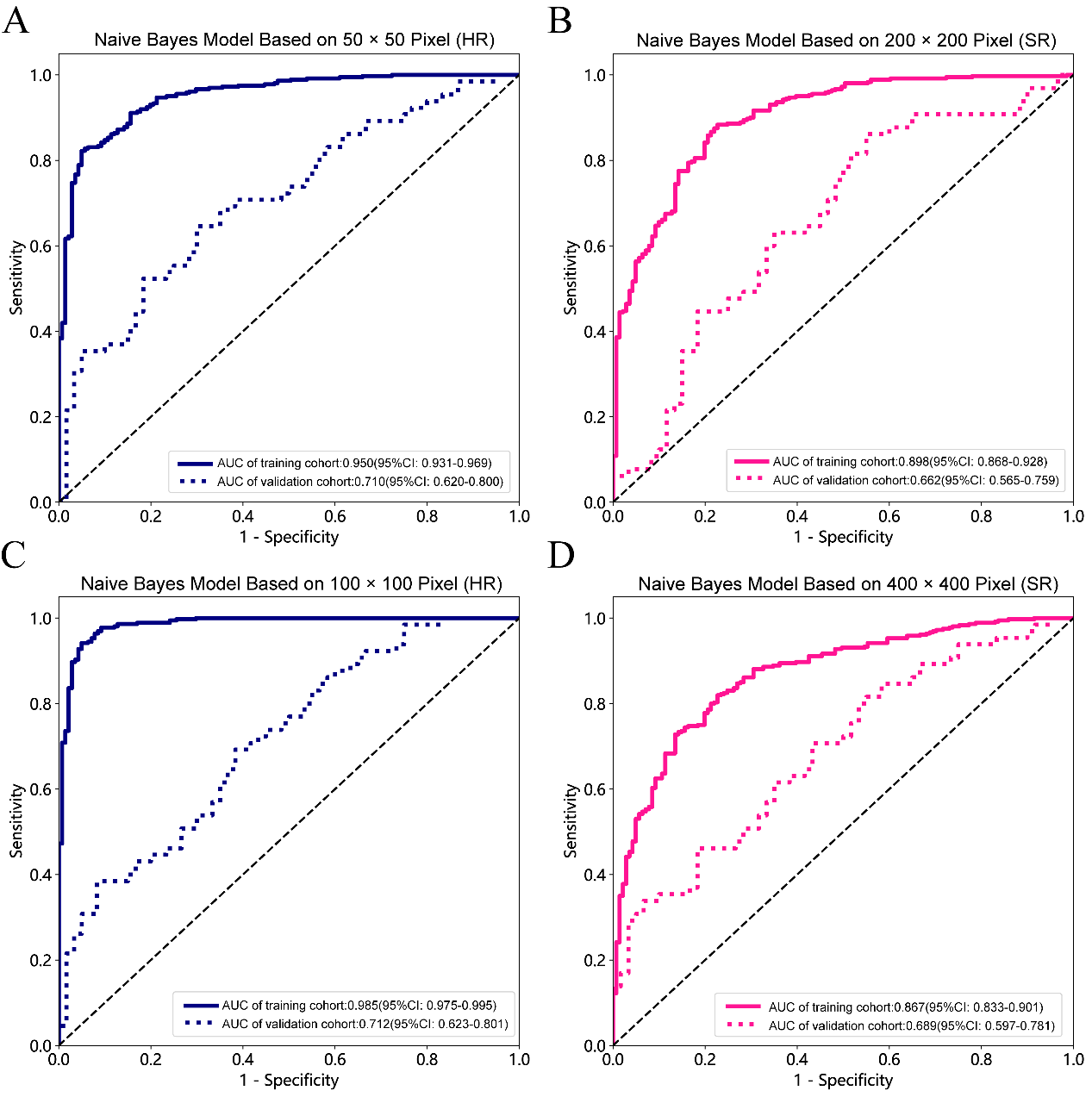


AUC: area under ROC curve; HR: high-resolution; SR: super-resolution.

**Supplementary Figure 2.** Receiver operating characteristic (ROC) curves of the different pixel in the Logistic Regression models (DenseNet201). A: 50 × 50 pixel images (HR). B: 200 × 200 pixel images (SR). C: 100 × 100 pixel images (HR). D: 400 × 400 pixel images (SR).


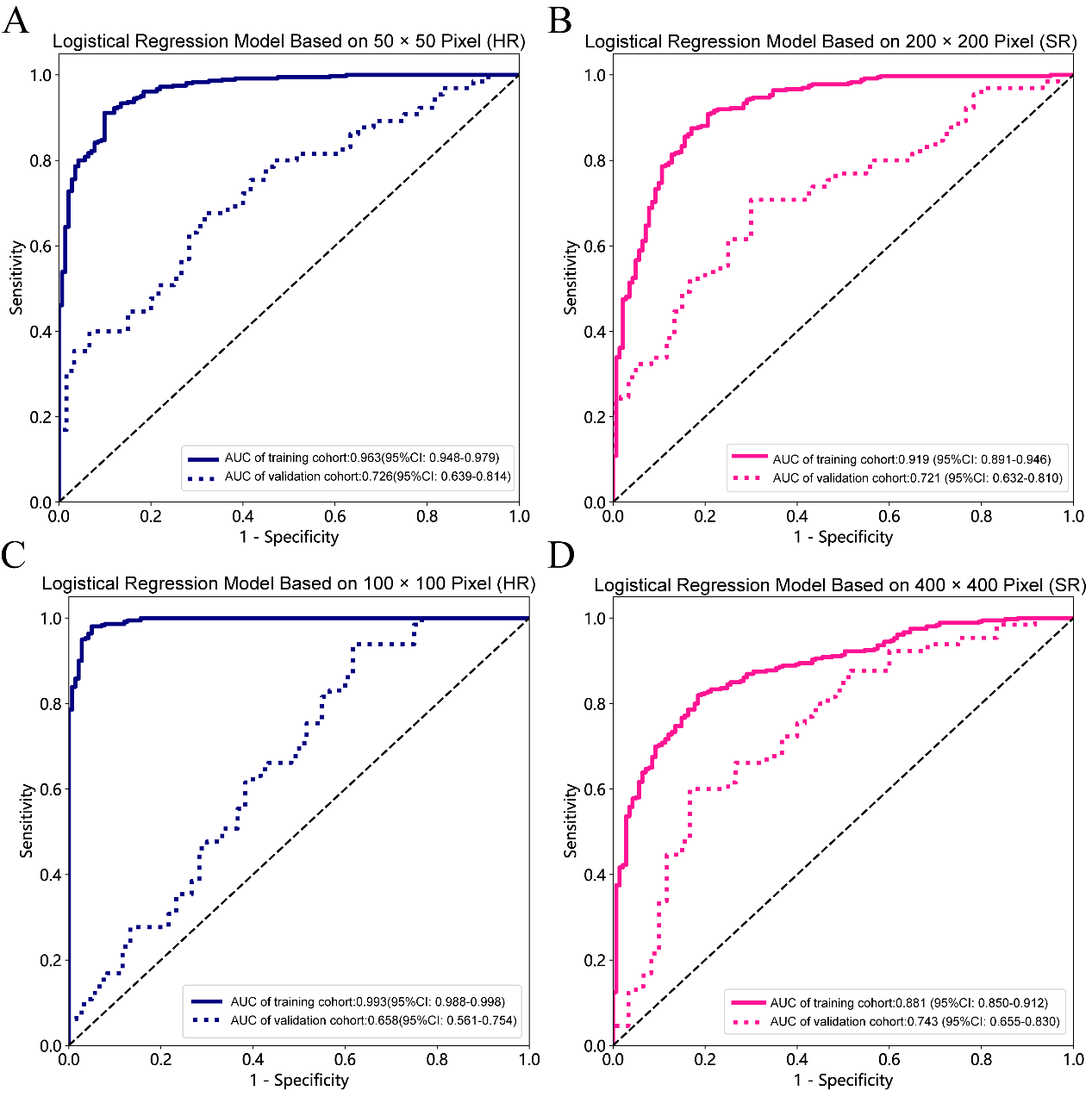


AUC: area under ROC curve; HR: high-resolution; SR: super-resolution.

**Supplementary Figure 3.** Receiver operating characteristic (ROC) curves of the different pixel in the Naive Bayes models (GoogLeNet). A: 50 × 50 pixel images (HR). B: 200 × 200 pixel images (SR). C: 100 × 100 pixel images (HR). D: 400 × 400 pixel images (SR).

**
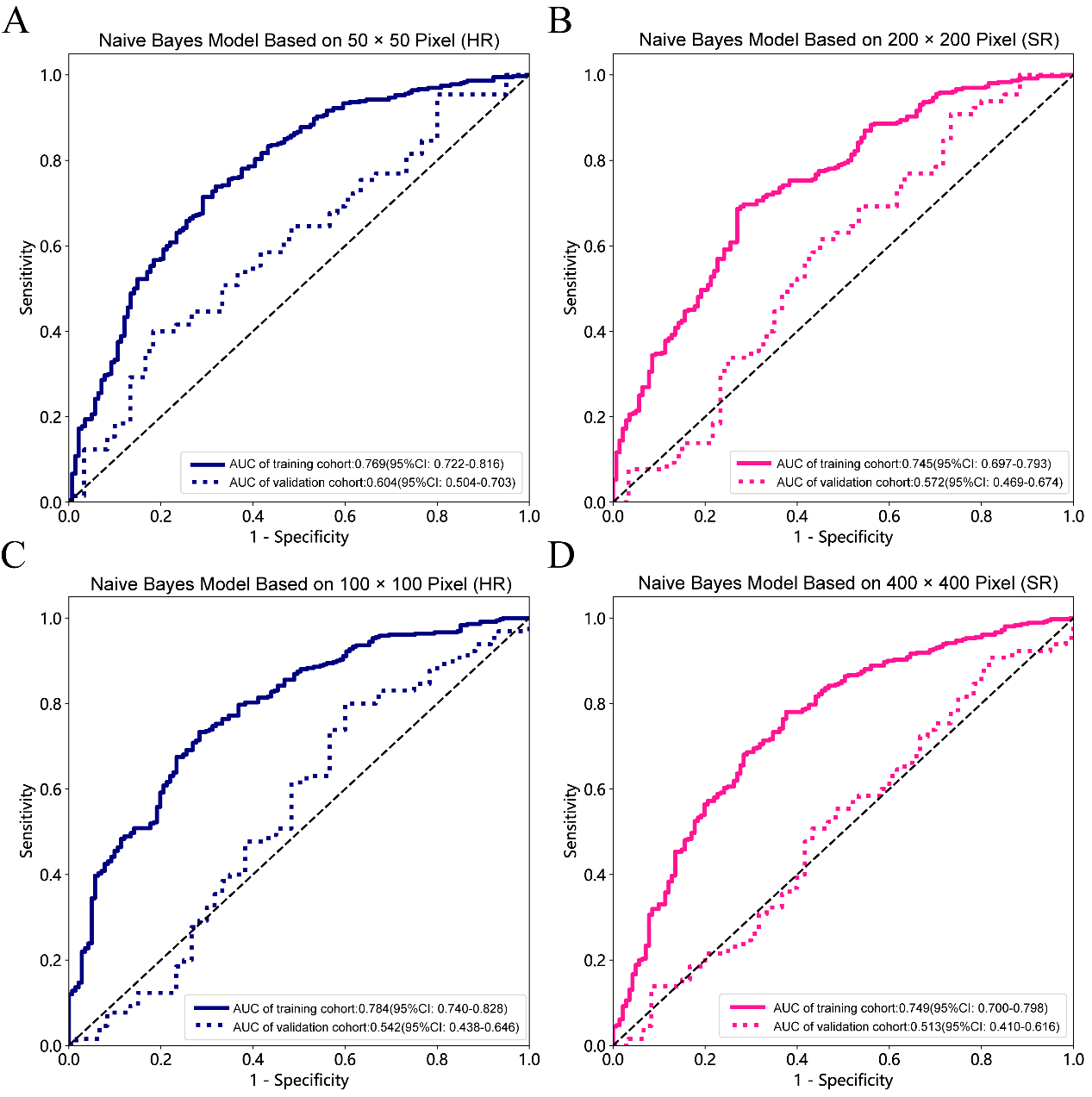
**

AUC: area under ROC curve; HR: high-resolution; SR: super-resolution.

**Supplementary Figure 4.** Receiver operating characteristic (ROC) curves of the different pixel in the Logistic Regression models (GoogLeNet). A: 50 × 50 pixel images (HR). B: 200 × 200 pixel images (SR). C: 100 × 100 pixel images (HR). D: 400 × 400 pixel images (SR).

**
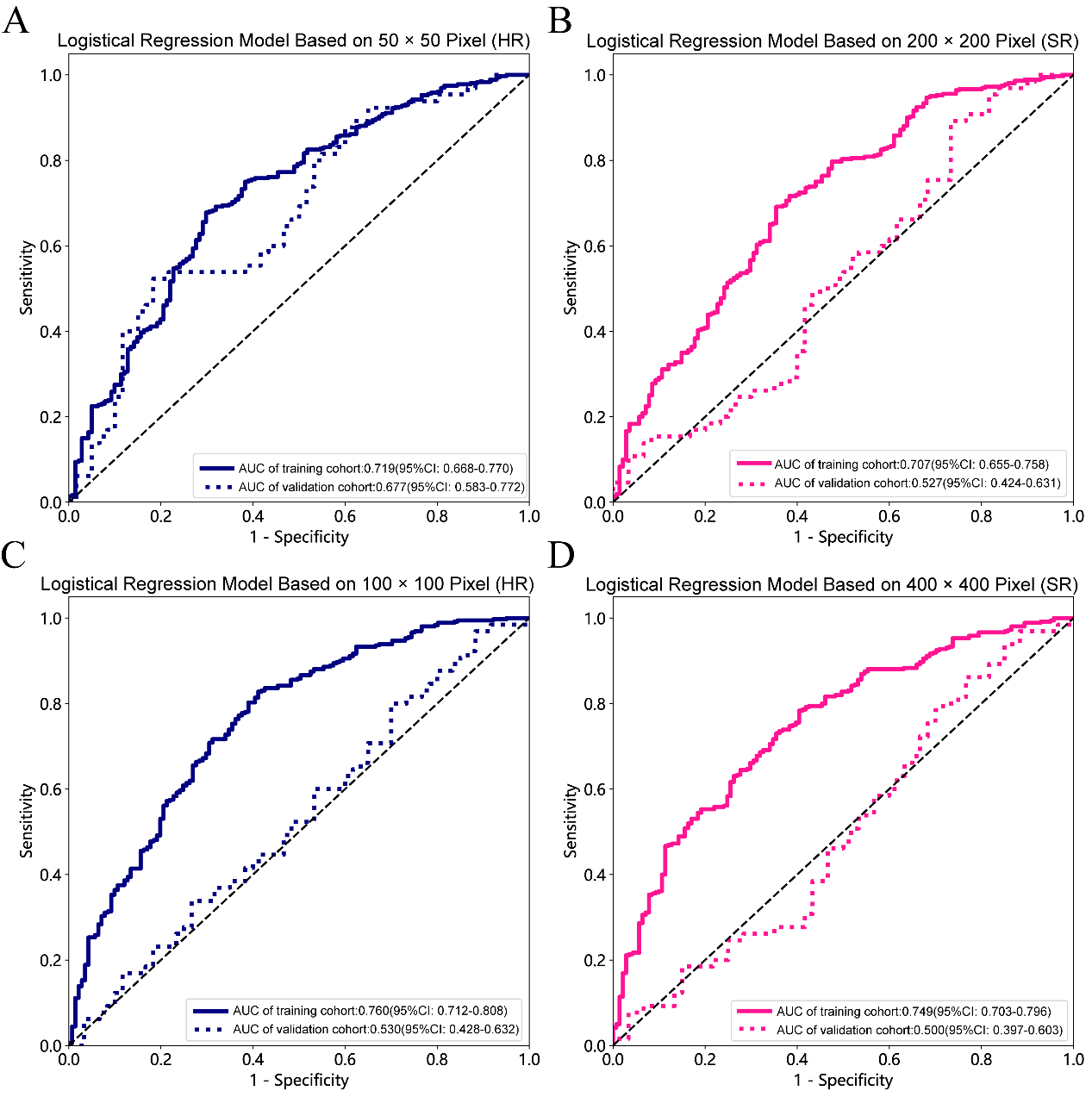
**

AUC: area under ROC curve; HR: high-resolution; SR: super-resolution.

**Supplementary Figure 5.** Confusion matrix and sample prediction histogram in the Naive Bayes models of different pixel images (ResNet152) (Mixed validation cohort). A: 50 × 50 pixel images (HR). B: 200 × 200 pixel images (SR). C: 100 × 100 pixel images (HR). D: 400 × 400 pixel images (SR).


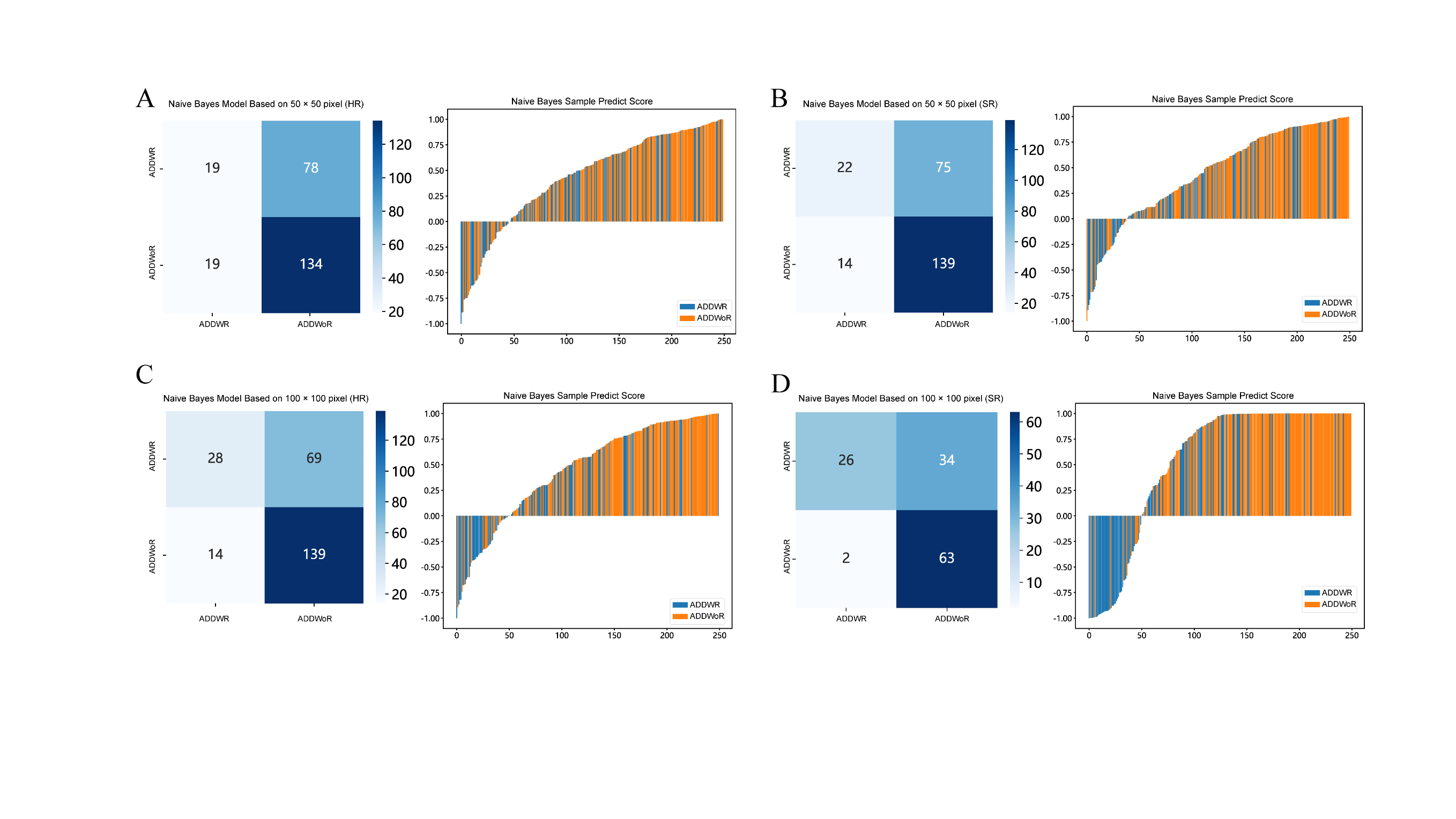


ADDWR: anterior disc displacement with reduction; ADDWoR: anterior disc displacement without reduction; HR: high-resolution; SR: super-resolution.

**Supplementary Figure 6.** Confusion matrix and sample prediction histogram in the Logistic Regression models of different pixel images (ResNet152) (Mixed validation cohort). A: 50 × 50 pixel images (HR). B: 200 × 200 pixel images (SR). C: 100 × 100 pixel images (HR). D: 400 × 400 pixel images (SR).


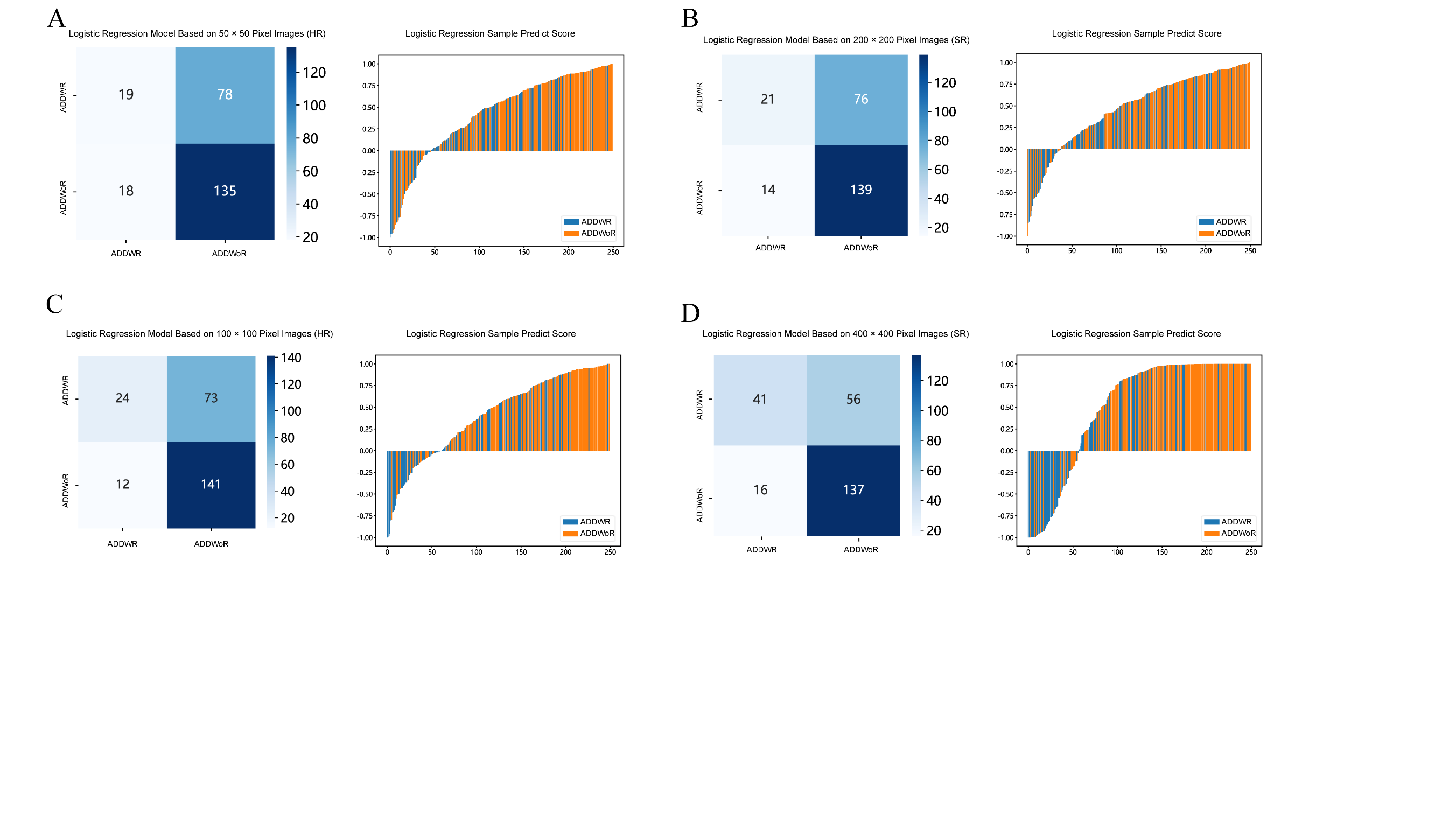


ADDWR: anterior disc displacement with reduction; ADDWoR: anterior disc displacement without reduction; HR: high-resolution; SR: super-resolution.
